# Amphoteric starch derivatives as reusable flocculant for heavy-metal removal†

**DOI:** 10.1039/c7ra12798g

**Published:** 2018-01-03

**Authors:** Liang Wu, Xingrong Zhang, Long Chen, Huan Zhang, Chengbi Li, Yin Lv, Yisheng Xu, Xin Jia, Yulin Shi, Xuhong Guo

**Affiliations:** Key Laboratory for Green Processing of Chemical Engineering of Xinjiang Bingtuan, School of Chemistry and Chemical Engineering, Shihezi University Shihezi 832003 P. R. China shiyulin@shzu.edu.cn; State Key Laboratory of Mineral Processing Beijing 102628 China; State Key Laboratory of Chemical Engineering, East China University of Science and Technology Shanghai 200237 China guoxuhong@ecust.edu.cn

## Abstract

A pH-responsive amphoteric starch derivative (PRAS) bearing dual functional groups (amino and carboxyl groups) was prepared through etherification of starch with 2-chloro-4,6-diglycino-[1,3,5]-triazine. PRAS exhibits a reversible pH-response property in aqueous solution. The attractive property of PRAS is that it could be used as an effective flocculant for heavy metal-ion (*e.g.* Cu(ii) and Zn(ii)) removal from wastewater by changing pH. The transition of hydrophobicity–hydrophilicity would produce shrinkage of the polymer matrix, facilitating the release of heavy-metal ions from the saturated flocculant. As an ideal flocculant PRAS displayed outstanding stability and reproducibility, whose remove rate for Cu(ii) and Zn(ii) remained at 93% and 91% after three flocculation/regeneration cycles.

## Abstract 2

A pH-responsive starch-based flocculants containing both cationic and anionic functional groups has been developed. The saturated flocculant can be facilely regenerated and separated from the solution by applying an external pH stimulus.

## Graphical abstract

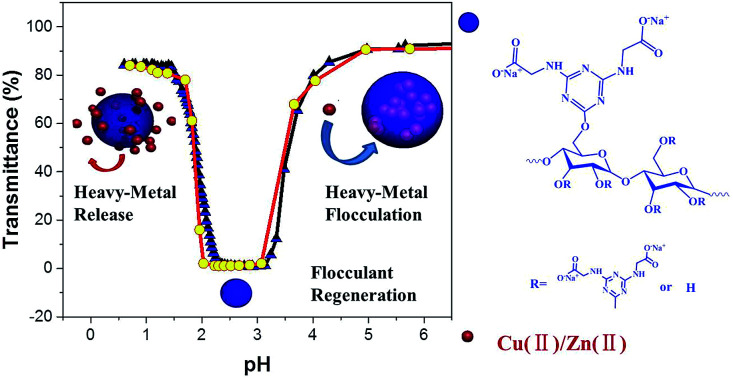

## 1 Introduction

Waste water containing heavy metals is often discharged by metal plating plants, mining smelting plants, and battery manufacturing plants, and has caused increasingly serious environmental problems.^1–3^ Zinc or copper is a trace element essential for human health, but excessive zinc or copper can trigger severe toxicological concerns and health problems.^4–6^

Adsorption and coagulation/flocculation processes have been extensively investigated to remove toxic heavy-metal ions from industrial effluents, and the latter has gained tremendous interest from industry due to their ability to enhance the formation of larger flocs and to promote solid–liquid separation of colloidal suspensions.^7–9^ Additionally, these flocculants have drawn particular attention as a water-soluble polymer due to their low cost (lower coagulant dose requirements), flexibility, simplicity, and easy operation.^10^

The main disadvantage of flocculation with water-soluble polymers is the difficulty in regeneration and reusability after water treatment.^11,12^ The flocculants characterized by both high removal efficiency and facile regeneration are expected in both fundamental and applicable studies. It is desirable to de-coagulate the heavy-metal ions and to restore the flocculant to a state similar to its initial properties. The flocculant regeneration stage is very important for decreasing the costs of the overall processing and for opening the possibility of recovery and further reuse of the heavy metals from the regenerated solution. However, the recovery of flocculant is challenging, and thus much less studied than the flocculation process. The difficulty of the recovery may due to the following reasons: (1) there exists high affinity between heavy metals and the flocculants including charge neutralization, polymer bridging, and electrostatic interactions. (2) Commercial flocculants are always highly soluble in water, and lack of controlled hydrophile-lipophile balance on the polymer chain which makes it hard to separate flocculant from the regenerated solution.

Stimulus-responsive materials offer advantages for intelligent separation that switch their affinity for the target substance on and off in response to physical or chemical changes from the external environment.^13–15^ Compared to the traditional materials, the special hydrophobicity-controlled polymers show significant advantages in both separation rate and efficiency. Moreover, stimuli-responsive polymers enable straightforward recycling of solids for effective reuse with undiminished target-substance uptake and no physical change or damage.^16^ Taking this into account, Cao *et al.* developed a temperature and pH dual-responsive oil/water separation material that could be used for highly controllable oil/water separation processes.^17^ This material enables quantitative capture and elution of oil by adjusting the temperature and pH without using organic solvents. Therefore, stimuli-responsive materials have high potential in water/oil separation.^18^

Traditional inorganic metal-based (polyaluminum chloride) and synthetic polymeric (polyacrylamide and its derivatives) flocculants are non-degradable, and directly discarding them after use will lead to secondary contamination of the environment.^19^ Over the last decade, environmentally friendly flocculants based on polysaccharides were developed.^19–22^ In our previous work,^11,12^ temperature- and/or pH-responsive starch derivatives were used to build smart starch-based flocculants with reversible phase transitions in an aqueous solution. The as-prepared materials can remove dye from dyeing effluent with high efficiency and easy recyclability. Furthermore, the utilized starch-based flocculants can be easily decomposed because of their biodegradable and eco-friendly characteristics.^9^

**Scheme 1:**
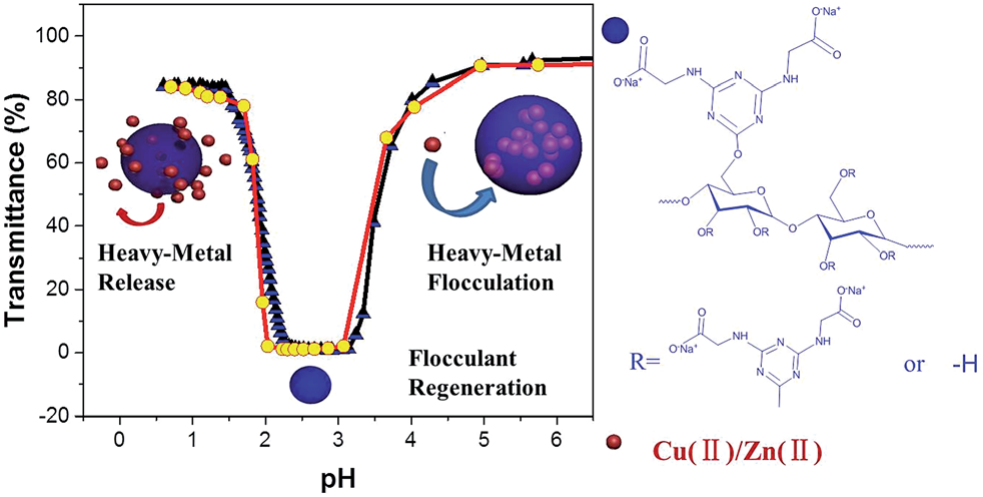
(a) Schematic representation of the cyclic flocculation/regeneration process; (b) molecular structure of the PRAS.

In this work, a pH-responsive starch derivative containing both cationic and anionic functional groups was developed through etherification of starch with 2-chloro-4,6-diglycino-[1,3,5]-triazine (CDT). The effects of the zwitterionic moiety and the hydrophobic moiety (*s*-triazine ring) on associative interactions were studied by utilizing turbidimetric titrations in aqueous media. The saturated flocculant based on pH-responsive amphoteric starch (PRAS) can be facilely regenerated and separated from the solution by changing pH (Scheme 1). The transition of hydrophobicity–hydrophilicity can produce a shrinkage of the polymer matrix, facilitating the release of heavy-metal ions from the flocculant. The de-flocculation and reusability were also investigated in terms of the flocculation performance after several flocculation/desorption cycles.

## 2 Experiment

### 2.1. Materials and reagents

Raw maize starch (ST, purity >98%, w/w) was obtained from Heng-hui Food Co., Ltd. (Xinjiang, China). Cyanuric chloride (purity 99%) was purchased from Tokyo Chemical Industry Co., Ltd. Glycine and ammonium hydroxide solution were obtained from Shanghai Tian Scientific Co., Ltd. Copper chloride [CuCl_2_·2H_2_O] (purity 99%) was provided by Fuchen Chemical Co., Ltd. (China). Zinc chloride (ZnCl_2_) (purity 98%) was obtained from Sinopharm Chemical Reagent Co., Ltd. (China). 2-Chloro-4,6-diglycino-[1,3,5]-triazine (CDT) was conducted according to the literature.^23^ The synthesis route of CDT is depicted in the ESI (Scheme 1S†). Distilled water was used throughout the entire flocculation/regeneration experiments. All the chemicals were used as-received without further purification.

### 2.2. Preparation of pH-responsive amphoteric starch

**Fig. 1:**
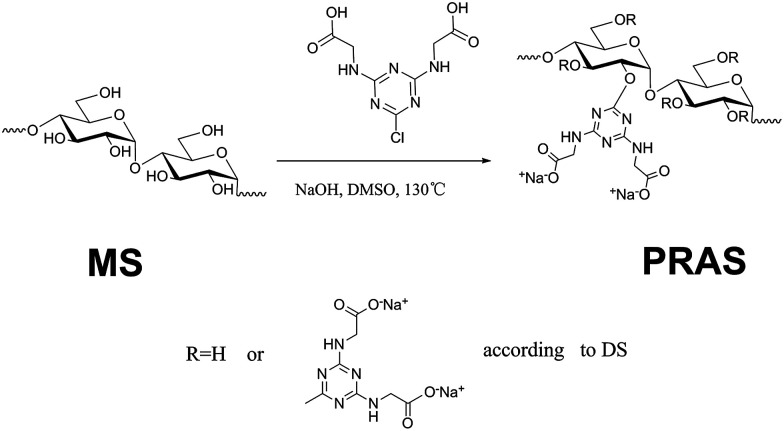
Synthesis of PRAS by etherifying reaction.

PRAS was prepared *via* an etherifying reaction between CDT and starch (ST) in dimethyl sulfoxide (DMSO). The synthesis of PRAS is shown in Fig. 1. Typically, ST (0.028 mol, 4.9 g), solid sodium hydroxide (0.196 mol, 8.2 g), CDT (0.084 mol, 22.0 g), and DMSO were mixed in a four-necked flask under a N_2_ stream. The mixture was then heated to 80 °C and maintained at that temperature for 20 min. Thereafter, the mixture was stirred at 130 °C for 10 h and then cooled to room temperature. Afterwards, three times the solution volume of methanol were added to the content of the flask, and the resulting precipitate was separated by filtration and then dried in a drying oven at 105 °C for 10 h. The product was purified by dialysis molecular weight cut-off (MWCO) 7000 Da against distilled water for 96 h, followed by lyophilization. Then PRAS (DS = 0.17) was obtained. Preparation method for the PRAS with the DS = 0.23 is detailed in the ESI (Text S2†).

The degree of substitution (DS) of hydroxyl groups replaced by CDT groups can be calculated according to eqn (1), where the nitrogen amount (*N*) is determined by elemental analysis. The determination of C, H and N contents in PRAS were measured by German Element Vario EL III. Operating condition was oxidation furnace temperature 1150 °C, reduction furnace temperature 850 °C and column temperature 110 °C under a O_2_ stream for 30 min. The parameter 162 represents the molar quantity of the repeating unit in ST, and 226 is the molar quantity of CDT group residues on the carbohydrate side chains.1
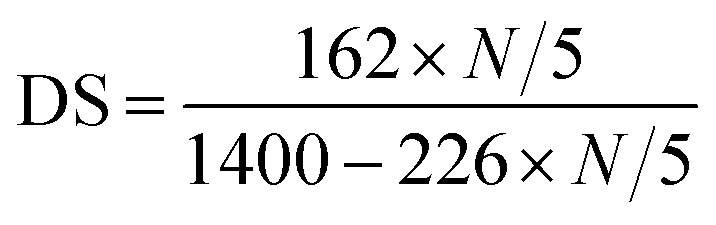


### 2.3. Characterization of PRAS

Infrared spectra of ST and PRAS were recognized with a Fourier-transform infrared spectroscopy (FTIR) spectrometer (JASCO IR-430, Japan) within a wave number range of 400–4000 cm^−1^ at a resolution of ±2 cm^−1^ by means of the KBr dispersion method. The samples were prepared by dry basis. ^1^H nuclear-magnetic-resonance (^1^H NMR) spectra were obtained using a 400 MHz spectrometer (Varian INOVA 400, USA) after the 5–10 mg purified samples were dissolved in 0.5 mL of NaOD/D_2_O. C, H, and N elemental analysis of PRAS was performed using an elemental analyzer (Vario EL, Germany). The zeta potential (ZP) was measured by a Micromeritics (USA) zeta/nanoparticle analyzer. In aqueous medium, a series of 2 g L^−1^ PRAS (DS = 0.23) solution with setting up pH values at room temperature were prepared. The samples were carried out by flow cell with 90° optical source.

### 2.4. pH responsiveness of PRAS

The solution properties of PRAS were evaluated by turbidity measurements according to the literature.^24–26^ PRAS (DS = 0.17 and 0.23) powder (500 mg) was mixed with deionized water (50 mL), then, 5, 10, and 25 mL of 10 g L^−1^ PRAS solutions were pipetted into 50 mL water to prepare 1, 2, and 5 g L^−1^ PRAS solutions (initial pH value 8), respectively. The optical transmittance of the PRAS solution was determined at 590 nm using an ultraviolet-visible (UV-vis) spectrophotometer with a glass cell. The recovery curve was measured in two steps. Firstly, by dropwise adding 1 M HCl to 2 g L^−1^ PRAS (DS = 0.23) solution and recording the corresponding change in the transmittance in the pH range 1.0–8.0 at room temperature. Secondly, the transmittance change of PRAS solution was carried out in repeated cycles by adding 1 M NaOH. Moreover, the ZP value of PRAS was measured under different pH conditions.

### 2.5. Flocculation experiments

In all metal flocculation experiments, standard jar tests were conducted at room temperature. Before each test, a 10 g L^−1^ PRAS stock solution was prepared by dissolving PRAS powder (0.5 g) in distilled water (50 mL).

CuCl_2_·2H_2_O was dissolved in distilled water to yield a 3.9 g L^−1^ Cu(ii) stock solution and adjusted pH to 10.1 by NH_3_·H_2_O. The Zn(ii) stock solution (3.7 g L^−1^, pH 9.3) was prepared and stored in the similar way. In a typical flocculation, distilled water (9.0 mL) and 0.2 mL of Cu(ii) or Zn(ii) stock solution were transferred into a centrifuge tube (15 mL). After a half hour, 0.05 mL PRAS stock solution was added to the simulative wastewater. Then the pH of the simulative wastewater was adjusted to the appropriate level by the addition of 1 M HCl or NaOH, after which water was added to obtain a total volume of 10 mL. In this system, the final concentrations of PRAS, Cu(ii) and Zn(ii) were 50, 78 and 74 mg L^−1^, respectively.

The jar test was repeated three times, corresponding results are analyzed with average values and std. deviation.^27^ The resulting mixture was shaken at (a) 150 rpm for 2 min, (b) 50 rpm for 5 min, and (c) 30 min of precipitate without stirring at room temperature by using constant temperature shaker (Blue Pard THZ-98A, China) according to the literature.^10^ Subsequently, the precipitate was kept still for 30 min to allow settling, and then the filtrate gathered by filtration using filter paper (0.45 μm) for further residual concentration (RC) measurements. The appropriate dilution ratio was determined from preliminary tests and the RC of contaminants by using an atomic absorption spectrophotometer (AAS, Shimadzu AA-6300, Japan). All glassware was prepared by immersing it overnight in dilute HNO_3_, then washed by distilled water. Using an air-acetylene flame as a flame atomizer, the height of burner and the air acetylene ratio were 8 mm and 6 : 1, respectively. Atomic absorption measurements were made at a hollow cathode lamp of 324.7 (Cu) and 213.8 (Zn) nm. Determination of Cu(ii) and Zn(ii) in diluted filtrate by AAS.

The residual concentration of heavy metals was calculated from the calibration curve (Fig. S1†). Contaminant removal (*R*%) and the metal flocculation capacity were calculated from the following equations:^28^2
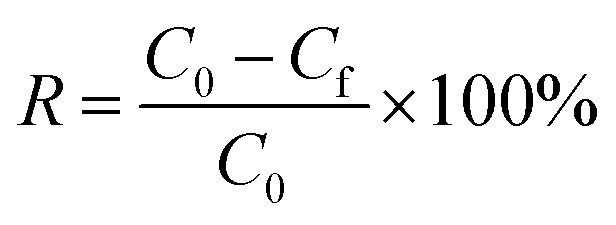
where *C*_0_ and *C*_f_ (mg L^−1^) denote the initial and equilibrium contaminant concentration in the filtrate, respectively.

### 2.6. Regeneration of PRAS

To study the reusability of PRAS for removal of metal(ii), 6 mL of Zn(ii) or Cu(ii) stock solution, and distilled water (590 mL) were transferred into a beaker (1 L). After a half hour, 1.2 mL PRAS stock solution was added to the simulative wastewater. Then the pH of the mixed solution was adjusted to 8.0, followed by the use of the mixing stages for flocculation experiments.

After the first flocculation, the flocs were settled by centrifugation step of 3652 × *g* (Kaida TG16G, China) for 10 min and 10 mL of the clarified layer of solution at a depth of 2 cm was collected. And then heavy metals in the solutions were determined by AAS. After cleaning and washing the flocs, the reusability of PRAS was studied. The flocs were totally dissolved in 10 mL water at pH 1.5. It was ensured that the most of metal ions were released and that the polymer networks were stretched by the protonation of amino groups. PRAS was then regenerated accompanied by increasing elution solution pH to 3.0. After the removal of regenerated flocculant from the solution by centrifugation. Cu(ii) and Zn(ii) in the supernatant liquid were also determined by AAS. The regenerated flocculant was again tested for further flocculation of metal ions. Flocculation and deflocculation experiments were conducted for four cycles as described above.

## 3 Results and discussion

### 3.1. Characterization

#### 3.1.1. FTIR spectra

**Fig. 2:**
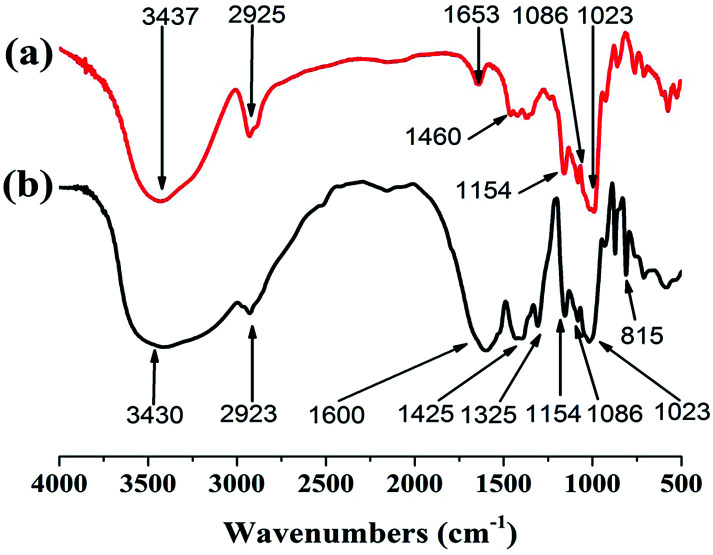
FTIR spectra of (a) ST and (b) PRAS with DS = 0.17.

Two kinds of samples (DS 0.17 and 0.23) were synthesized and used as flocculants throughout the experiment. The FTIR spectra of ST and PRAS with DS = 0.23 were recorded and shown in Fig. 2. In the spectrum of ST (Fig. 2a), the characteristic peaks at 3437, 2925 cm^−1^ and a triplet peak at 1154 cm^−1^ correspond to O–H,^12^ C–H stretching vibrations in the methylene groups, and C–O–C stretching vibration of the glucose rings of ST,^29^ respectively. The wavenumber at 1653 cm^−1^ was assigned as C–O–C stretching vibrations, while the peaks of C–H bending vibrations (1460 cm^−1^) in the methylene groups of starch was detected.^29,30^ Absorption bands appeared at 1154, 1086 and 1023 cm^−1^ are the characteristic peaks of the glucose rings of ST.^11,31^

As illustrated in Fig. 2b, the new peaks at 1600, 1425 and 1325 cm^−1^ can be assigned to the –COO^−^ stretching vibration in PRAS synthesis.^31,32^ The new peak at 1600 and 1425 cm^−1^ comes from the stretching vibrations of the *s*-triazine ring in comparison to ST.^8,11^

#### 3.1.2. ^1^H NMR spectra

**Fig. 3:**
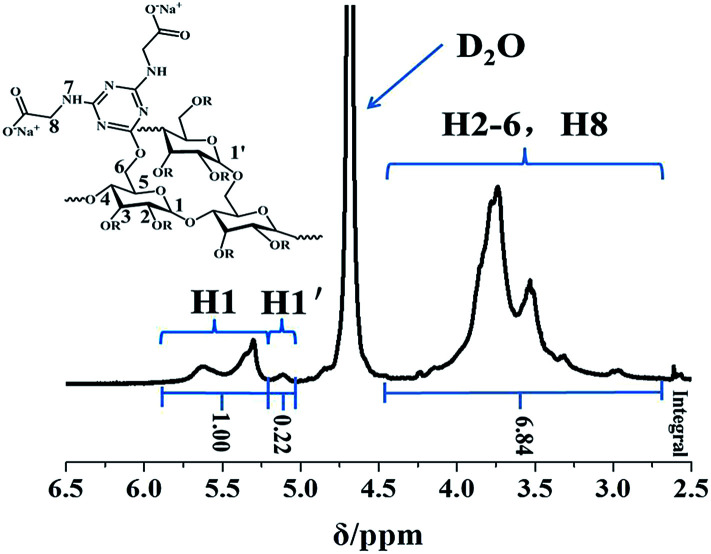
^1^H NMR spectra of PRAS (DS = 0.17) in deuterated NaOD/D_2_O.

^1^H NMR spectra of PRAS and the corresponding assignments are shown in Fig. 3. The proton of the anhydroglucose unit (AGU) in corn starch is readily observed using ^1^H NMR (NaOD/D_2_O). The detailed assignments are as follows: *δ* = 5.30 [H1, (1–4)-α-linkages], and *δ* = (H2–H6), which is consistent to a previous study.^33,34^ The anomeric proton (H1) of the unmodified anhydrous glucose unit (AGU) appeared at 5.20 ppm.^35^ The chemical modification of the AGU then led to a significant shift of the corresponding signals.^35^ Furthermore, proton signals, *δ* = 3.80 (H8), at the methylene group of –CH_2_COO^−^ are observed in the ^1^H NMR spectra. The FTIR and ^1^H NMR results directly indicate that the CDT moieties are incorporated within the ST network after chemical modification.

### 3.2. pH responsiveness properties of PRAS

#### 3.2.1. DS-induced phase transition

**Fig. 4:**
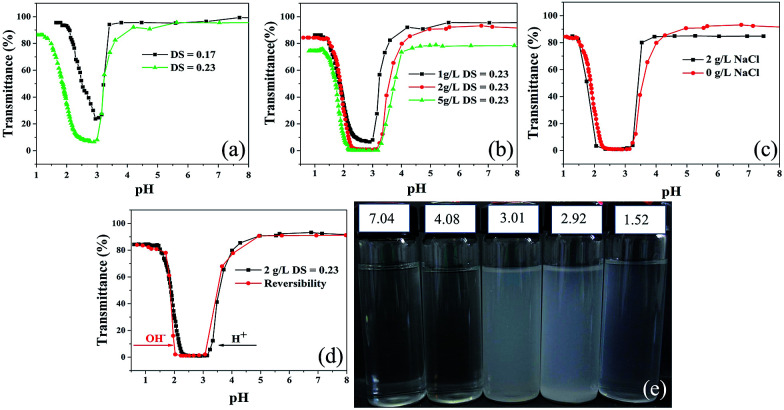
Transmittance *vs.* pH of aqueous PRAS under absorbance at 590 nm for (a) 1 g L^−1^ PRAS aqueous solution with different DS. (b) PRAS (DS = 0.23) at different concentrations. (c) 2 g L^−1^ PRAS (DS = 0.23) aqueous solution with different NaCl concentrations. (d) Reversible phase transition of the 2 g L^−1^ PRAS (DS = 0.23) aqueous solution by the addition of acid in the black line and base in the red line. (e) Photographs of 1 g L^−1^ PRAS (DS = 0.23) aqueous solution at various pH values.

As shown in Fig. 4a, the changes in transmittance of PRAS were determined based on transmittance measurements at a wavelength of 590 nm over a range of pH values from 1 to 8. The U-shaped curve of PRAS became more obvious with the increase of the degree of substitution (DS). Upon changing DS of PRAS, the ratio of hydrophobic and hydrophilic groups in a polymer chain was changed, which altered the relatively sharp transition of turbidity with pH. As an amphoteric polyelectrolyte, aqueous PRAS solution tends to precipitate around the isoelectric point (IEP).^10,36^ As for PRAS with DS 0.23, three relatively sharp pH phase transitions (pH ≈ 4.0, 3.0, and 1.5) were observed during reducing pH by addition of HCl. When the pH was higher than 4.5, the transmittance reached a plateau and kept unchanged owing to the deprotonation of the carboxyl groups.^25^ With the decrease of pH value from 4.5 to 3.0, the clear solution turned cloudy due to the protonation of hydrophilic carboxyl groups. Further decreasing pH from 3.0 to 1.5, the turbid solution became clear again, indicating the existence of protonated amine groups.

#### 3.2.2. Concentration-induced phase transition

As shown in Fig. 4b, the transmittance of PRAS solution decreased with increasing PRAS concentration (DS = 0.23) at the same pH. The pH-responsive properties of PRAS can be described by the critical pH (pH*), at which the transmittance is decreased by 50% in a manner similar to the lower critical solution temperature (LCST).^26,37^ In Fig. 4b, the pH* are 3.7, 3.5, and 3.2 for concentrations of 5.0, 2.0, and 1.0 g L^−1^, respectively. These results indicated that pH* increased with increasing polymer concentration. Such fact verifies that higher concentration is correlated with some degree of increased interchain interactions and with sufficient polymer aggregate formation at higher pH.^38^

#### 3.2.3. Ionic strength effect on phase transition

The effect of ionic strength was also investigated regarding to the phase-transition behavior of PRAS (DS = 0.23) as shown in Fig. 4c. The U-shaped distribution of PRAS solutions without NaCl is narrower than that with 2 g L^−1^ NaCl. Upon increasing ionic strength, electrostatic interactions among the protonated carboxyl groups were shielded gradually. Moreover, as has been reported,^39^ water molecules could be polarized by the adjacent anions, leading to weak intermolecular hydrogen bonding. Thus, the hydrodynamic volume and the hydrophilic interaction of the polymer chains decreased.^40,41^ As a result of reduction in interactions among PRAS chains, the phase transition became more insensitive to pH at higher ionic strength.

As shown in Fig. 4d, the pH-induced phase transition was also stable and reversible with addition of 1 M NaOH or HCl. Owing to the pH responsiveness of PRAS, as-prepared flocculant should be able to selectively capture and release heavy-metal ions from wastewater by adjusting the pH of the solution.

#### 3.2.4. Reversible phase transition

As was shown in Fig. 4d, the pH-induced and reversible phase transition behavior is stable and reversible with the addition of 1 M NaOH or HCl solutions. It is proven that PRAS has reversible phase transition behavior that is based on the data obtained. When PRAS dissolved in the aqueous or in crystal, it was ampholyte that existed as a sort of zwitterions or dipole ionic with different pH. The ionization balance is expressed as follows:^42^3
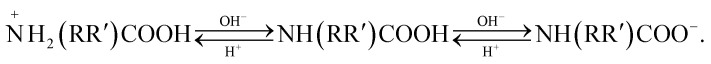


As-prepared flocculant can selectively capture and release heavy metal ion from waste water by adjusting the pH of solution.

### 3.3. Removal of metal(ii) from aqueous solution

#### 3.3.1. pH-dependent removal of metal(ii) from water solution

**Fig. 5:**
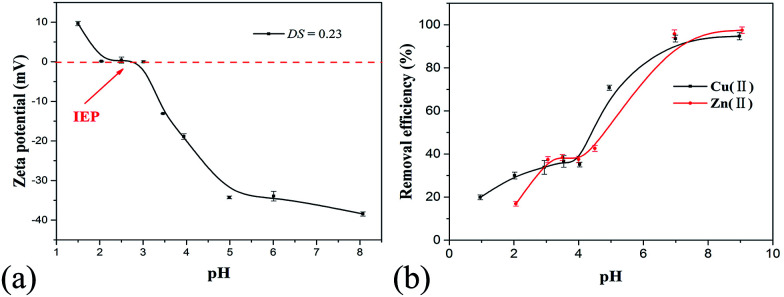
(a) ZP as a function of pH for 2 g L^−1^ PRAS (DS = 0.23) solution at 25 °C, and (b) effect of pH on the removal efficiency of Cu(ii) and Zn(ii) by PRAS.

The pH-dependence of zeta potential (ZP) of PRAS (DS = 0.23) was examined as shown in Fig. 5a. PRAS is composed of both cationic and anionic functional groups. The ZP-pH profiles indicate that the IEP of PRAS is very close to the midpoint in the pH range of 1.5 to 4.0 at which the cationic charge just balances the anionic charge on the PRAS. Below pH 1.5 or above pH 4, the zwitterionic polymer re-dissolved to form a transparent solution. With the increase of the amount of hydroxide ions (pH), the equilibrium shifted to the right, and the ZP reduced and became negative (Fig. 5a).

The metal-removal efficiency of PRAS solution (50 mg L^−1^) for Cu(ii) (78 mg L^−1^) and Zn(ii) (74 mg L^−1^) at various pH values is presented in Fig. 5b. Upon increasing pH of the flocculation solution, the flocculation performance enhanced. The optimum pH region of PRAS to remove Cu(ii) and Zn(ii) was over 7.0, where the ZP closed to the negative maximum (Fig. 5a). Upon increasing pH value, the carboxylic groups on the PRAS were progressively deprotonated and reached fully dissociation, while the flocculant was dissolved in water because of the high ionizability of the anionic groups, and formed a more extended conformation. Polymer bridging and charge neutralization took place between the negatively charged flocculant and heavy-metal cation, leading to increased flocculation efficiency.^9,43^ When pH value approached to the IEP (pH = 3.0), the flocculant was transformed to insoluble material at this critical pH. At this stage, the ability of charge neutralization was weakened due to the offset of the charges between cationic and anionic functional groups.

#### 3.3.2. Effect of flocculant dosage on flocculation

**Fig. 6:**
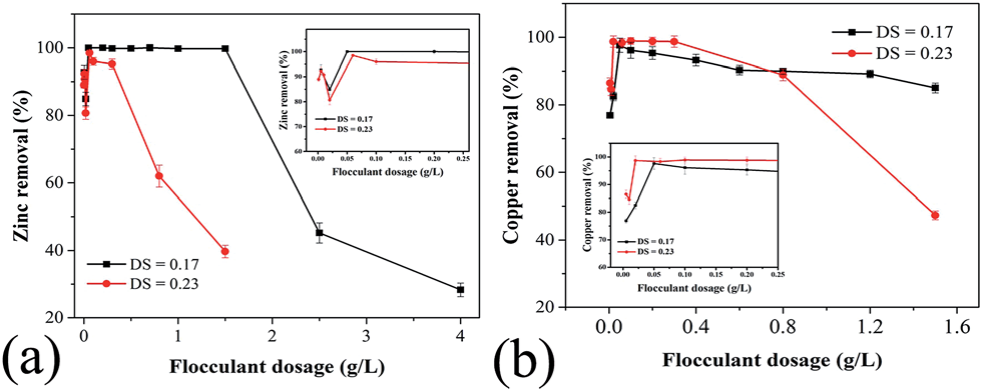
Effect of PRAS dosage on (a) Zn(ii) and (b) Cu(ii) removal efficiency. The changing parameter is the degree of substitution (DS).

To determine the optimal PRAS dosage for metal ion removal, the effect of flocculant dosage on flocculation was investigated. Amphoteric starch products with DS of 0.17 and 0.23 were exposed to an aqueous solution containing 78 mg L^−1^ Cu(ii) and 74 mg L^−1^ Zn(ii). The results were shown in Fig. 6.

Zn(ii) removal efficiency increased and reached almost 100% upon increasing the PRAS dosage to 0.07 g L^−1^. At low dosages, the number of anionic charges was insufficient for complete neutralization of the cationic charges of metal ions, resulting in weak destabilization of colloids by the flocculant. The maximum Zn(ii) removal appeared at the amphoteric starch dosages around 0.07 g L^−1^. However, excessive dosage makes the metal particles potentially completely covered by the absorbed polymer layer. Thus, steric repulsion generated well-established particles and resulted in a reduced efficiency.^9,44–46^ In addition, PRAS has triazine rings with large π-electron clouds, which cause an enhanced coordination with metal ions.^8^ For highly substituted amphoteric starch derivative (DS = 0.23), the Zn(ii) removal efficiency became more sensitive to PRAS dosage, and the window for the best Zn(ii) removal efficiency reduced significantly (Fig. 6a). Similar results can be observed in Fig. 6b, which shows the effect of amphoteric starch dosages on Cu(ii) removal.

### 3.4. Regeneration of PRAS

PRAS is a promising flocculant for the removal of Cu(ii) and Zn(ii) from waste water. Owing to the pH responsiveness of PRAS, the PRAS can be simply regenerated by adjusting the pH of the elution solution.

The flocs were totally dissolved in water at pH 1.5. This ensured that the metal ions were almost fully released. Then, PRAS was regenerated by increasing the elution solution pH to 3.0. After the removal regenerated flocculant from the solution by centrifugation, the solution was analyzed using AAS.

**Fig. 7:**
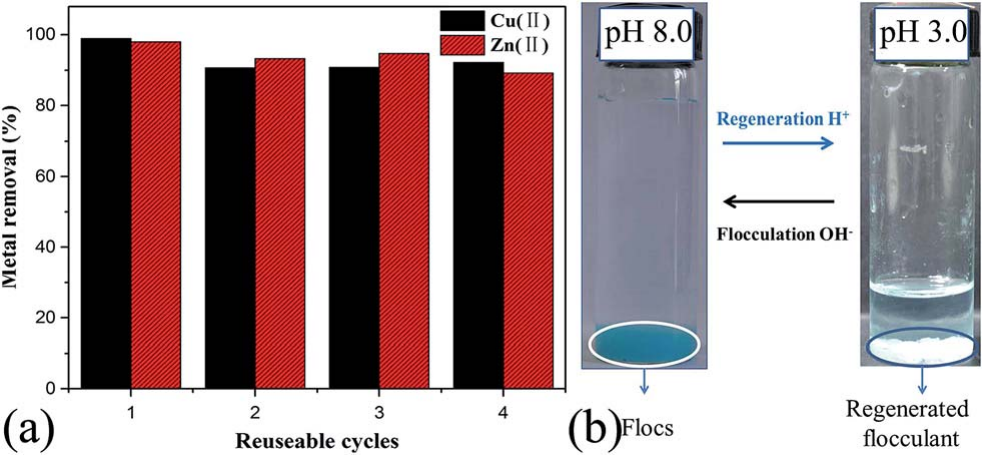
(a) Metal removal efficiency of PRAS in four successive cycles of flocculation-regeneration. (b) Photographs of regenerated elution of Cu(ii) before and after regeneration by PRAS.

In addition, the stability of the PRAS and the reproducibility of the flocculation properties were also evaluated after several flocculation/regeneration cycles. As shown in Fig. 7, the removal efficiency in the fourth cycle was up to 92% and 89% for copper and zinc, respectively, which means after three cycles PRAS retained 93% and 91% remove rate for Cu(ii) and Zn(ii), respectively. The reuse of PRAS in thus reduced the costs of the overall flocculation process significantly.

## 4 Conclusions

In summary, a novel pH-responsive amphoteric starch derivative (PRAS) bearing dual functional groups (amino and carboxyl groups) was successfully prepared through etherification of starch with 2-chloro-4,6-diglycino-[1,3,5]-triazine (CDT). PRAS exhibited reversible pH-response properties in aqueous solutions. Cu(ii) and Zn(ii) ions can be almost fully removed by PRAS from contaminated water through a flocculation process. Owing to its pH responsiveness, the subsequent reusability of PRAS could be achieved by adjusting the solution pH. In addition, the stability of the PRAS and the reproducibility of the flocculation properties were also evaluated after several flocculation/regeneration cycles. The results showed that PRAS retained 93% and 91% remove rate for Cu(ii) and Zn(ii), respectively, after three cycles.

## Conflicts of interest

There are no conflicts to declare.

## Supplementary Material

RA-008-C7RA12798G-s001
