# Supplementary material for: Amphoteric starch derivatives as reusable flocculant for heavy-metal removal
Source: RSC Adv. 2018 Jan 3;8(3):1274–80. doi: 10.1039/c7ra12798g (PMC9077004; doi:10.1039/c7ra12798g)
Supplement: RA-008-C7RA12798G-s001 [file RA-008-C7RA12798G-s001.pdf]

Electronic Supplementary Material (ESI) for RSC Advances.
This journal is © The Royal Society of Chemistry 2017

Supplementary Information

Amphoteric Starch Derivatives as Reusable Flocculant for Heavy-Metal Removal

Liang Wu[a], Long Chen[a], Xingrong Zhang[b], Huan Zhang[a], Chengbi Li[b], Yin Lv[a], Tingting Wei[a], Yisheng Xu[a,c], Xin Jia[a], Yulin Shi[a]*, Xuhong Guo[a,c]*

[a]*Key Laboratory for Green Processing of Chemical Engineering of Xinjiang Bingtuan, School of Chemistry and Chemical Engineering, Shihezi University, Shihezi 832003, P.R. China.*

[b]*State Key Laboratory of Mineral Processing, Beijin 102628, China.*

[c]*State Key Laboratory of Chemical Engineering, East China University of Science and Technology, Shanghai 200237, China.*

Text S1. The synthesis route of 2-chloro-4,6-diglycino-1,3,5-triazine (CDT)

Cyanuric chloride (3.68 g, 20 mmol) in acetone (40 mL) was added dropwise to an ice-cold mixture of Na_2CO_3 (8.48 g, 80 mmol) and glycine (3.00 g, 40 mmol) in deionized water (60 mL) more than 30 min. The reaction mixture was stirred overnight at room temperature (RT). The reaction mixture was neutralized with concentrated HCl and filtered off. Then, the mixture washed three times with cold and deionized water and dried to get target product CDT(Khattab et al., 2016).

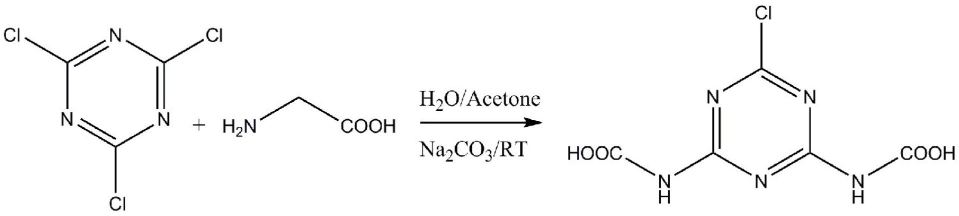

Scheme 1S. The synthesis route of CDT.

Text S2. Preparation of PRAS (*DS* = 0.23)

PRAS (*DS* = 0.23) was prepared via an etherifying reaction between CDT and starch(ST) in dimethyl sulfoxide (DMSO). Typically, ST (0.028 mol, 4.9 g), solid sodium hydroxide (0.253 mol, 10.7 g), CDT (0.115 mol, 30.2 g), and DMSO were mixed in a four-necked flask under a N_2 stream. The mixture was then heated to 80 °C and maintained at that temperature for 20 min. Thereafter, the mixture was stirred at 130 °C for 10 h and then cooled to room temperature. Afterwards, three times the solution volume of methanol were added to the content of the flask, and the resulting precipitate was separated by filtration and then dried in a drying oven at 105 °C for 10 h. The product was purified by dialysis molecular weight cut-off (MWCO) 7000 Da against distilled water for 96 h, followed by lyophilization.

Text S3. Standard curve

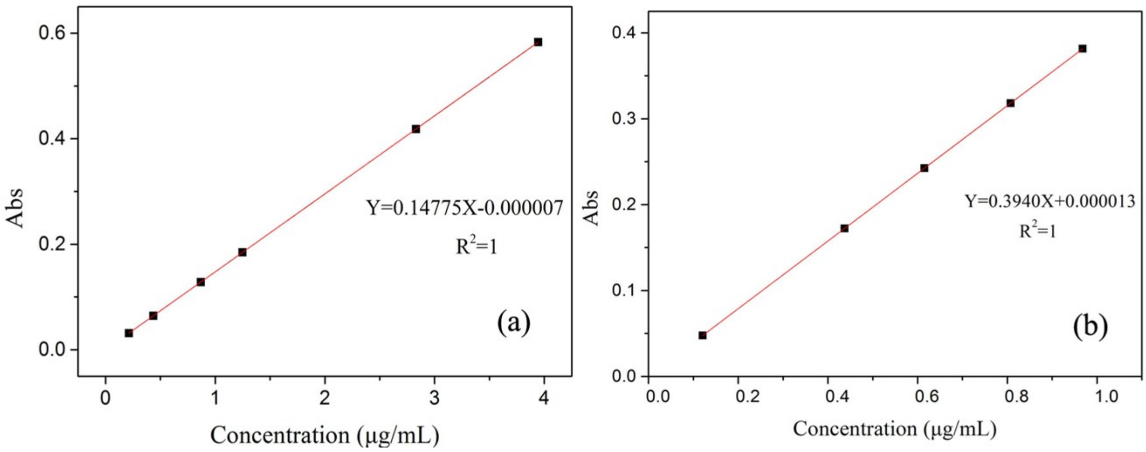

Fig. S1. Stadard curve of Znic (a) and Copper (b).

References

Khattab, S. N., Abdel Naim, S. E., El-Sayed, M., El Bardan, A. A., Elzoghby, A. O., Bekhit, A. A., & El-Faham, A. (2016). Design and synthesis of new s-triazine polymers and their application as nanoparticulate drug delivery systems. *New Journal of Chemistry*, 40(11), 9565-9578.
